# Differences in Mouse Maternal Care Behavior – Is There a Genetic Impact of the Glucocorticoid Receptor?

**DOI:** 10.1371/journal.pone.0019218

**Published:** 2011-04-28

**Authors:** Sabine Chourbaji, Carolin Hoyer, S. Helene Richter, Christiane Brandwein, Natascha Pfeiffer, Miriam A. Vogt, Barbara Vollmayr, Peter Gass

**Affiliations:** Central Institute of Mental Health, Mannheim, Germany; University of Chicago, United States of America

## Abstract

Depressive episodes are frequently preceded by stressful life events. Evidence from genetic association studies suggests a role for the glucocorticoid receptor (GR), an essential element in the regulation of stress responses, in the pathophysiology of the disorder. Since the stress response system is affected by pregnancy and postpartum-associated changes, it has also been implicated in the pathophysiology of postpartum depression. Using a 2×2 factorial design, we investigated whether a heterozygous deletion of GR would influence maternal care behavior in C57BL/6 and Balb/c mice, two inbred strains known to display qualitative differences in this behavior. Behavioral observation was carried out between postnatal days 1 and 7, followed by a pup retrieval test on postnatal days 7 or 8. While previously noted inter-strain differences were confirmed for different manifestations of caring behavior, self-maintenance and neglecting behaviors as well as the pup retrieval test, no strain-independent effect of the GR mutation was noted. However, an interaction between GR genotype and licking/grooming behavior was observed: it was down-regulated in heterozygous C57BL/6 mice to the level recorded for Balb/c mice. Home cage observation poses minimal disturbance of the dam and her litter as compared to more invasive assessments of dams' emotional behavior. This might be a reason for the absence of any overall effects of the GR mutation, particularly since GR heterozygous animals display a depressive-like phenotype under stressful conditions only. Still, the subtle effect we observed may point towards a role of GR in postpartum affective disorders.

## Introduction

Depression is an etiologically complex psychiatric disorder with both genetic and environmental factors impacting on disease vulnerability. Regarding the former, the glucocorticoid receptor (GR) is a pivotal candidate gene, and there is evidence for functionally and pathophysiologically relevant roles of GR single nucleotide polymorphisms from genetic association studies [1], [2]. Additionally, GR responsiveness can be influenced by epigenetic mechanisms induced by known risk factors for depression [3]. Among those, stress is a central player, and the majority of major depressive disorder first episodes are preceded by stressful life events [4]. With their high emotional salience, pregnancy and childbirth are stressful life events for many women that may cause significant anxiety and discomfort [5], [6]. The stress response system undergoes numerous changes during pregnancy and postpartum [7] and has accordingly been implicated in the pathophysiology of postpartum depression [8], [9], a disorder affecting approximately one in seven women worldwide [10]. With reduced ability to perceive a child's signals, to interpret them correctly and react accordingly, as well as lesser degree of playful body contacts and loving interactions [11], [12], bonding between mother and child as well as childcare can be seriously disturbed in depressed women [13].

Different strategies of targeted mutagenesis in mice have been employed to study the effects of GR dysfunction and its potential behavioral consequences [14]. Animals carrying abnormalities in the GR (nervous-system/forebrain-specific or heterozygous knockout) are more susceptible to develop an emotional or depressive-like phenotype [15]. While forebrain-specific knockout mice display a depression-like phenotype in adulthood under basal conditions, heterozygous GR mice possess a heightened vulnerability that manifests only when animals are exposed to challenging situations through a specific external stressor such as restraint- or footshock stress [16], [17], [18]. In consideration of these findings, we were interested to find out if disrupted glucocorticoid signaling via a heterozygous GR deletion, a depression-prone genetic make-up, induces immediate differences in caring behavior of mice towards their offspring in a way reminiscent of the phenomenology of postpartum depression. We hypothesized the emergence of a depression-like behavioral profile in GR heterozygous animals, specifically an increase in maternal neglect and/or a reduction of caring behaviors.

Balb/c and C57BL/6 mice, two of the most commonly used inbred strains of laboratory mice, are known to differ markedly in emotional reactivity, anxiety-like behavior and general activity with Balb/c mice being more reactive and fearful than C57BL/6 mice [19], [20], [21]. Moreover, they differ with respect to the maternal care delivered to their offspring: C57BL/6 dams have been found to spend more time licking and grooming their pups than their Balb/c conspecifics [22], [23], [24], [25], [26], which generates a considerable early-life environmental condition, a fact that has to be borne in mind with regard to any kind of developmental hypothesis [27]. These inter-strain differences have been employed to study the relevance of early-life adversity and different genetic variations associated with an increased risk for affective and anxiety disorders [23], [28], [29], [30], [31]. However, interest has mainly focused on offspring effects while largely ignoring the impact of these variations on maternal care behavior.

Variation in the genetic background of strains is known to affect test outcomes [32]. In addition, strain differences, which are – direct or indirect – genetic differences [33], are tied to differences regarding maternal care. Hence, in order to determine the external validity of the behavioral phenotypes while at the same time revealing biologically relevant interactions [34], [35], we intended to pursue our research objective with a 2×2 factorial design [36]. This allows for the discrimination of effects that generalize across strains and those depending on an interaction of strain and GR genotype.

## Materials and Methods

### Animals

According to a 2×2 factorial design we investigated a total number of 26 mature dams of two inbred strains (C57BL/6N, Balb/c) that either carry a heterozygous mutation of the GR or not: C57BL/6N wildtypes (+/+; n = 6), C57BL/6N with a heterozygous mutation of the GR (+/−; n = 7), Balb/c wildtypes (+/+; n = 7), and Balb/c with a heterozygous mutation of the GR (+/−; n = 6). Mating was conducted by introducing two females into the cage of a male wildtype conspecific. Delivery was dependent on mating success but took place within a period of 2–3 weeks. Litters were left intact, delivery cages remained unchanged during the observation period. Since not enough females became pregnant during the first mating session, additional time-delayed matings were necessary to reach a statistically representative number of animals. Thus, behavioral observations were conducted in two independent replicates, each comprising 13 dams. Animals were constantly kept in conventional type III cages (length 42 cm×width 25 cm×height 14,5 cm) in a regular day-night cycle (lights on at 6:00, lights off at 18:00) with nesting material (tissue), and food and water *ad libitum*.

### Ethics Statement

All procedures complied with the regulations covering animal experimentation within the EU (European Communities Council Directive 86/609/EEC) and Germany (Deutsches Tierschutzgesetz). Experiments (breeding, mating, handling and observations) were approved by the German animal welfare authorities (Regierungspräsidium Karlsruhe, permit number 35-9185.81/G144/04) and performed in the animal facilities of the Central Institute of Mental Health in Mannheim, Germany. Moreover, all efforts were made to minimize the number of animals used and the severity of procedures applied in this study.

### Generation of GR heterozygous animals

The founders of GR-heterozygous animals were developed as previously described by Tronche [37]. Briefly, by employing homologous recombination in embryonic stem cells, a modified GR allele was generated with a loss of exon 3, encoding the first zinc finger of the receptor's DNA-binding domain, and a subsequent shift of the open reading frame. GR-heterozygous animals carrying this “null-allele” had been backcrossed for more then 10 generations into the C57BL/6 background as described earlier [17]. For the present study, this deficient GR-allele was also backcrossed for more then 10 generations into the Balb/c strain.

### Behavioral observation

All behavioral observations were carried out by three experienced researchers throughout the study. The observers entered the room at least five minutes before the start time of the observation to allow the mice to habituate to their presence. Observations were carried out in two sessions per day between postnatal days 1 and 7. Behavioral observation sessions took place between 9:00 and 10:30 and between 14:00 and 15:30, when white lights were on and animals were in their inactive phase. As recording method we used scan sampling throughout the experiment [38], scanning the behavior of each dam one after another. Thereby, the observer recorded whether or not a specific behavior occurred on the instant of each sample point (instantaneous sampling). Each session lasted about 45 min, yielding 30 scans per session and dam. Scan data were then summed up across session and day and related to the total amount of recorded scans. Dams were observed for 5 out of 7 days, leading to a total number of 270 to 330 scans per dam. To avoid a bias in maternal care related to observation day, the five observation days were randomly chosen across PND 1 and PND 7. Behaviors were manually recorded onto check sheets.

Behavioral analysis was performed on the basis of previously employed ethological parameters (Table 1). In nest behaviors comprised: ‘licking/grooming’, ‘active nursing’, ‘passive nursing’ and ‘nest building’ as manifestations of caring behavior; ‘self-grooming’ as a manifestation of dam self-maintenance; and ‘pups out of nest’ as a neglecting behavior that related neither to the dam's immediate self-care nor her interacting with her litter. Out-of-nest behaviors were ‘eating/drinking’ and ‘self-grooming out of nest’ as self-maintenance behaviors and ‘climbing/digging’ as manifestation of neglecting behavior.

**Table 1:** Ethogram used for the assessment of maternal care behavior in C57BL/6N and Balb/c dams.

	Description	Categorization
**In nest**		
licking/grooming	dam touches the pup's body with her tongue, dam handles the pup's body with her forepaws or nose	caring behavior
active nursing	dam presents an upright dorsal arch posture with the depressed head posture over the pups which are attached to the nipples	caring behavior
passive nursing	dam lies immobile on pups and has her eyes open or closed	caring behavior
nest building	dam collects and/or handles nesting material around the pups with mouth or forepaws	caring behavior
self-grooming	licking, brushing or scratching fur or paws with tongue or paws inside the nest	self-maintenance
pups out of nest	pups are outside of the nest with no contact to dam, to other pups or nesting material	neglecting behavior
**Out of nest**		
eating/drinking	chewing food, sawdust or feces; licking water from bottle tip	self-maintenance
self-grooming	licking, brushing or scratching fur or paws with tongue or paws outside the nest	self-maintenance
climbing/digging	dam climbs with all four paws attached to the cage lid, dam uses forepaws to scratch sawdust away from her body	neglecting behavior

Behavioral measures are categorized according to their presumed function in ‘self-maintenance’, ‘caring’ and ‘neglecting behavior’.

### Pup retrieval test

Behavioral observations were completed by a pup retrieval test [39], [40]. This test was performed on PND 7 or 8 depending on the motility of the pups, which was very sensitive to change during specifically this period because the time of delivery on PND 0 certainly varied. Latencies were recorded until a mother: a) retracted two of her pups, which had been placed in the corners of the home cage, back into the nest (‘back in nest’) and b) crouched over the litter (‘crouching over pups’). There was a cut-off after 300 seconds if the dam did not retrieve her pups. Moreover, the amount of time was recorded that the dam spent handling her pups (‘handling’).

After this procedure the entire observation series was terminated, and mother and pups were placed into a fresh cage.

### Statistical evaluation

All data were analyzed using General Linear Models (GLM). To meet the assumptions of parametric analysis, residuals were graphically examined for homoscedasticity and outliers, and, when necessary, raw data were transformed using square-root or angular transformations. Based on the 2×2 factorial design, a two-way ANOVA with “strain” and “GR genotype” as between-subject factors was used to analyze maternal care behavior. For the analysis of the pup retrieval test, we included “PND” as a blocking factor in the statistical design in order to account for variation due to the testing day. Where significant interactions occurred, t-tests were calculated following the GLM to obtain more specific information on where differences are. To address the problem of multiple comparisons we then used Holm's Sequential Bonferroni Procedure [41] to adjust alpha-levels. All statistical tests were conducted using the software package SPSS/PASW (version 18.0 for Windows). Differences were considered to be significant at p<0.05.

## Results

### Behavioral observation

**Figure 1:**
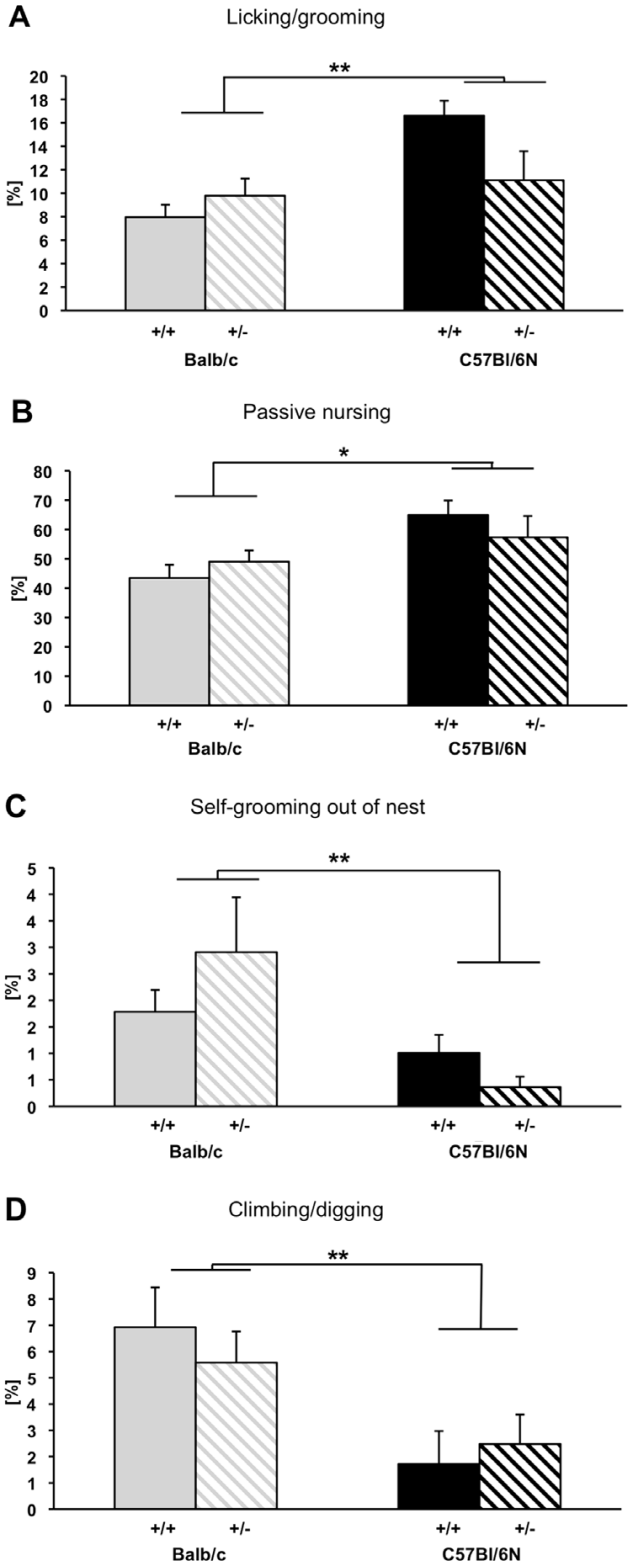
Effects of strain and genotype on maternal care behavior. Behavioral strain differences between C57BL/6N and Balb/c mothers with a glucocorticoid receptor wildtype (GR +/+) or a heterozygous deletion (GR +/−) are exemplarily presented for four different behavioral measures: (A) ‘licking/grooming’, (B) ‘passive nursing’, (C) ‘self-grooming out of nest’ and (D) ‘climbing/digging’. While strains were found to differ significantly in all four measures, GR genotype did not affect the behavior. Moreover, a significant strain-by-genotype-interaction was found with respect to ‘licking/grooming’. While C57BL/6N +/+ dams spent more time ‘licking/grooming’ than Balb/c mothers of both GR genotypes, no difference was found between C57BL/6N +/− mothers and Balb/c dams. Data are presented as untransformed means ± standard error of the mean, * p<0.05, ** p<0.01.

The analysis of maternal care behavior revealed an effect of strain on all three behavioral categories studied, while an effect of GR genotype on maternal behavior was not detected (Table 2). With respect to caring behavior, C57BL/6N mice displayed significantly more ‘licking/grooming’ (F_1,22_ = 8.469, p = 0.008, Fig. 1A) and ‘passive nursing’ (F_1,22_ = 7.365, p = 0.013, Fig. 1B) than Balb/c mothers. Furthermore, statistically significant strain differences occurred for self-maintenance behavior, demonstrating that Balb/c mothers spent more time ‘self-grooming out of nest’ than C57BL/6N dams (F_1,22_ = 12.120, p = 0.002, Fig. 1C). Concerning neglecting behavior, statistical analysis revealed a significant strain effect on ‘climbing/digging’ with Balb/c mice showing more ‘climbing/digging’ than their C57BL/6N conspecifics (F_1,22_ = 10.281, p = 0.004, Fig. 1D). Moreover, Balb/c pups tended to be outside the nest more often than C57BL/6N pups (F_1,22_ = 3.229, p = 0.086), indicating that Balb/c mothers were less observant of their litter. All other behavioral measures, including ‘active nursing’, ‘nest-building’, ‘self-grooming’, ‘feeding’ and ‘sleeping out of nest’ were not affected by strain (Table 2).

**Table 2:** Summary of all data generated by behavioral observations and the pup retrieval test.

	Balb/c GR +/+	Balb/c GR +/−	C57Bl/6N GR +/+	C57Bl/6N GR +/−	Transf	Strain	Genotype	Interaction	
**In nest**									
licking/grooming [%]	7,951±1,070	9,790±1,454	16,615±1,273	11,101±2,476	NT	0,008 **		0,043 *	
active nursing [%]	14,613±4,955	9,913±3,589	6,130±2,323	11,800±3,654	sqrt				
passive nursing [%]	43,471±4,482	49,027±3,801	64,870±4,993	57,299±7,275	NT	0,013 *			
nest building [%]	6,624±1,089	6,058±1,317	5,532±0,857	10,346±3,220	sqrt				
self-grooming [%]	4,517±0,579	6,140±1,710	3,848±0,863	5,304±1,214	sqrt				
pups out of nest [%]	5,507±2,484	2,070±1,485	1,498±1,265	0,246±0,123	sqrt	0,086 T			
**Out of nest**									
eating/drinking [%]	10,387±2,299	10,835±1,664	14,422±3,166	10,067±3,808	NT				
self-grooming [%]	1,783±0,410	2,908±1,037	1,010±0,338	0,360±0,198	sqrt	0,002 **			
climbing/digging [%]	6,924±1,514	5,578±1,186	1,718±1,254	2,477±1,123	NT	0,004 **			
**Pup retrieval test**									
handling [%]	26,410±4,661	24,658±5,226	34,614±8,825	47,402±6,456	NT	0,048 *			
pups back in nest [s]	165,286±35,313	135,600±35,730	107,167±33,491	83,000±22,290	angular				
crouching over pups [s]	232,714±33,622	166,600±39,829	178,500±41,680	206,143±44,680	angular			0,051 T	

Data analysis was done using GLMs on the basis of 26 dams belonging to four different treatment groups: Balb/c wildtypes (+/+; n = 7), Balb/c with a heterozygous mutation of the GR (+/−; n = 6 for behavioral observations, n = 5 for the pup retrieval test) C57BL/6N wildtypes (+/+; n = 6), C57BL/6N with a heterozygous mutation of the GR (+/−; n = 7). Data are given as untransformed means ± standard error of the mean (s.e.m.). The statistical analysis is summarized with respect to the transformation used (NT = not transformed, sqrt = square root transformation, angular = angular transformation) and the effects of “strain”, “GR genotype” and “strain-by-GR-genotype-interaction” (T = tendency; *p<0.05, **p<0.01).

Although statistical analysis did not reveal any direct effects of GR genotype on maternal care behavior, a strain-by-GR-genotype-interaction was found for ‘licking/grooming’ (F1,22 = 4.601, p = 0.043). Subsequent group comparisons showed that C57BL/6N +/+ mothers spent more time ‘licking/grooming’ than Balb/c +/+ (T11 = −5.253, p<0.001, significant after Bonferroni correction, Fig. 1A) and Balb/c +/− dams (T10 = −3.532, p = 0.005, significant after Bonferroni correction, Fig. 1A). Furthermore, no difference occurred between C57BL/6N +/− and Balb/c +/− mothers (T11 = −0.436, p = 0.671, Fig. 1A), and C57BL/6N +/− and Balb/c +/+ dams (T12 = −1.168, p = 0.266, Fig. 1A), indicating that the heterozygous mutation of the GR down-regulated ‘licking/grooming’ in C57BL/6N dams to the level of Balb/c mothers.

### Pup retrieval test

**Figure 2:**
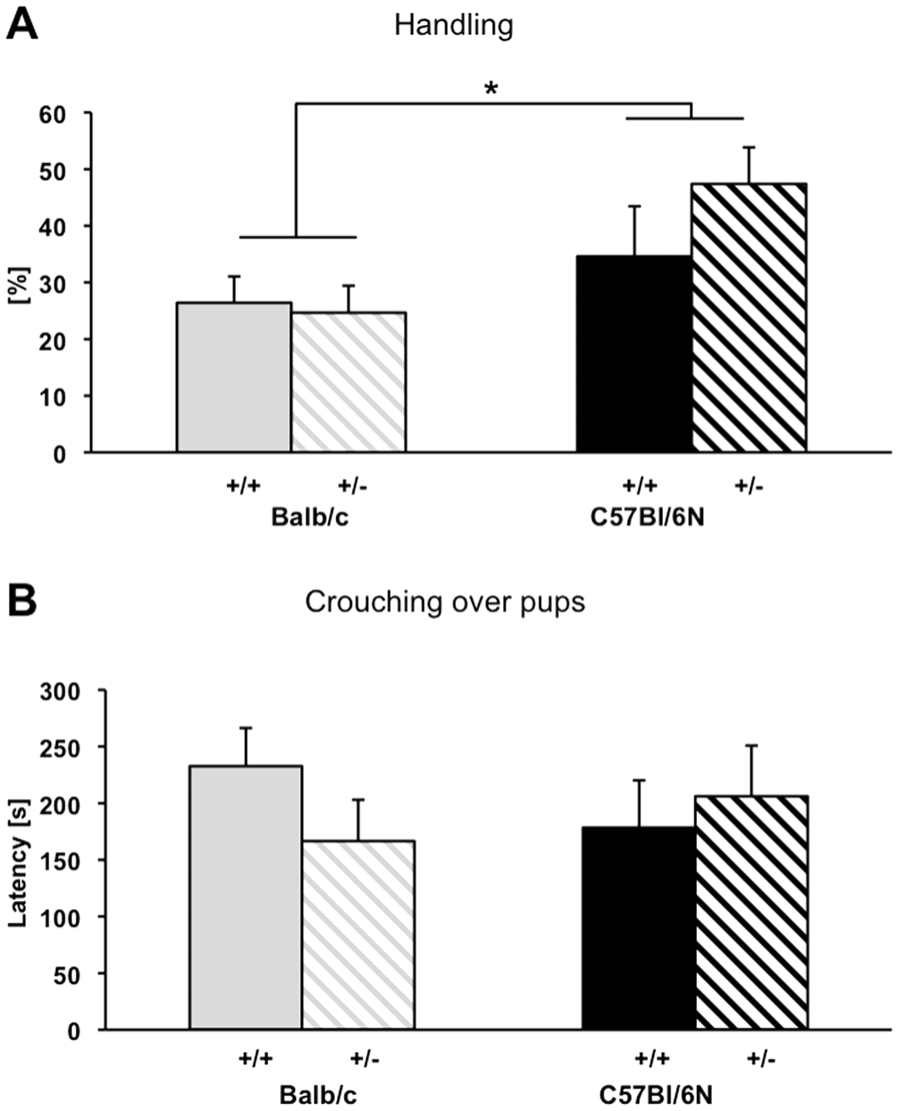
Effects of strain and genotype on maternal behavior in the pup retrieval test. Behavioral strain differences between C57BL/6N and Balb/c mothers with a glucocorticoid receptor wildtype (GR +/+) or a heterozygous deletion (GR +/−) are presented for two out of three behavioral measures: (A) ‘handling’ and (B) ‘crouching over pups’. Strains differed significantly in ‘handling’, but GR genotype did not affect any of the behavioral measures. However, a tendency for a strain-by-genotype-interaction was found with respect to ‘crouching’. Data are presented as untransformed means ± standard error of the mean, T p<0.1, * p<0.05.

When assessing the time it took for the dam to recollect two of her pups from the opposite corner of the home cage into the nest at PND 7 or PND 8, respectively, no differences were found between mothers of different strains and GR genotypes (Table 2). However, C57BL/6N mice were found to spend more time ‘handling’ their pups than Balb/c mothers (F_1,20_ = 4.450, p = 0.048, Fig. 2A). Furthermore, a strain-by-GR-genotype interaction tendency was found with respect to the behavioral measure ‘crouching over pups’ (F_1,22_ = 4.326, p = 0.051, Fig. 2B).

## Discussion

The objective of our study was to investigate the role and relevance of a heterozygous mutation of the glucocorticoid receptor in shaping maternal care in two strains of mice which have been described to exhibit differences in this type of behavior. The 2×2 factorial design enabled us to segregate the effects of the GR mutation that are potentially generalizable across strains from strain-related differences as well as interactions of GR genotype and strain. In light of the stress-induced proneness to depression of GR heterozygous animals and multiple HPA axis alterations during pregnancy and postpartum, we expected GR heterozygous mothers to display lower-quality maternal care than wildtypes.

We were able to confirm previously noted inter-strain differences in maternal care behavior in C57BL/6N and Balb/c mice. C57BL/6N dams demonstrated increased attendance inside the nest in closer contact to the pups as they spent more time licking and grooming. In addition, Balb/c mothers performed significantly more time climbing and digging out of the nest, i.e. a “neglecting” behavior without immediate relevance to the dams' own or the progeny's survival, as well as self-grooming outside the nest. Finally, in the pup retrieval test, C57BL/6N mothers spent a larger proportion of the test time handling their pups. While no overall independent GR genotype effect could be detected, an interaction between GR genotype and strain was observed in licking/grooming behavior whereby heterozygous C57BL/6N mothers' behavior was down-regulated to the level of Balb/c dams. The markedly reduced level of maternal care shown by that strain, however, might mask potential effects of the GR mutation taking the form of a further decrease of maternal care, which might be at odds with the minimum requirement to ensure pups' survival.

Since observation was carried out during the light, i.e. the animals' inactive, phase, one might ask for the influence of time of day, or activity phase, on the assessed behavior. Maternal care in mice and rats displays a circadian rhythm with the amount of time spent nursing higher during the light phase [42]. We thus decided to focus our observations on this more relevant phase.

Home cage observation as an experimental technique to assess the dams' behavior is completely non-invasive. This might be one reason why no strain-independent GR genotype-associated differences in maternal behavior were found in our experimental setting. Recalling previous studies of GR heterozygotes, these mice do not show a behavioral phenotype under basal, i.e. unstressed, conditions but only when exposed to a stressful challenge [17]. Hence, it is likely that more invasive manipulations of the postnatal environment could evoke a more pronounced depressive-like phenotype in GR heterozygous mothers. However, experimental modes using stress to trigger such a phenotype would necessarily induce maternal separation, which in and of itself is known to affect the offspring [43], which in turn is mediated by altered amounts of maternal licking [44], [45]. In order to further elucidate this matter, it would be necessary to separate the immediate ethological investigation of maternal care from that relating to depressive-like behavior in the mothers e.g. through application of Porsolt's Forced Swim Test or Learned Helplessness.

Our results provide an interesting avenue for further investigation into the role of GR disturbances in dams' postnatal emotional behavior. With an – albeit limited and subtle – interactive effect of the GR mutation and strain on maternal care, the phenotype is reminiscent of that of human postnatal depression where mothers impaired childcare and neglect often accompany binding difficulties the mothers experience towards their children [46]. Peripartal HPA axis changes have been investigated in both humans and animals, and there are few animal models of postpartum depression. The ovarian-steroid withdrawal model [47] is one model, but it does not allow for an exploration of offspring effects due to required ovariectomy in the females. There are models employing exposure to gestational stress [48] and postpartum treatment with high levels of corticosterone [49]. However, since gestational stress alters the amount of maternal licking, and maternal licking can affect the offspring's phenotype independent of the mother's emotional phenotype [48], [50], and handling of the dams for the corticosterone injections during postpartum [49] disturbs the litter, it is difficult to discriminate and clearly identify the relative effects and contributions of each variable in these paradigms.

The three categories employed to analyze maternal care behavior, i.e. ‘caring behavior’, ‘self-maintenance’ and ‘neglecting behavior’ differ from previously employed categorizations. Even though there are similarities with respect to the individual parameters assessed, these were grouped into different overarching categories. While Coutellier and colleagues differentiate ‘active nursing’, ‘passive nursing’ and ‘non-nursing/others’ [51], we considered it apt to establish an *a priori* categorization allowing for a more precise breakdown of non-nursing behaviors relevant for the condition of the litter in either a positive (self-maintenance) or negative (neglecting behavior) way. The category ‘caring behavior’ encompasses not only nursing *per se* but also activities like nest building [52], which, however, are of immediate importance for the offspring's welfare.

We assume our ethological method for monitoring the maternal environment by means of a non-invasive strategy will prove a valuable complementary tool to be employed in future studies regarding the effects of targeted mutagenesis in postpartum rodent emotional behavior and maternal care. Genetic and epigenetic GR modification with its behavioral consequences remains an interesting strategy for the further investigation of the pathophysiology of postpartum depressive disorder.
